# Editorial: The heart-brain connection in emotions, cognition, and dementia

**DOI:** 10.3389/fnagi.2022.1074280

**Published:** 2022-11-24

**Authors:** Knut A. Hestad, Knut Engedal, Ivana Hollan, Hélène Girouard

**Affiliations:** ^1^Department of Research, Innlandet Hospital Trust, Brumunddal, Norway; ^2^Department of Health and Nursing Science, Faculty of Health and Social Sciences, Inland Norway University of Applied Sciences, Elverum, Norway; ^3^Norwegian National Centre for Aging and Health, Vestfold Hospital Trust, Tønsberg, Norway; ^4^Department of Geriatric Medicine, Oslo University Hospital, Oslo, Norway; ^5^Norwegian University of Science and Technology, Gjøvik, Norway; ^6^Department of Pharmacology and Physiology, Faculty of Medicine, Université de Montréal, Montréal, QC, Canada; ^7^Groupe de Recherche Universitaire sur le Médicament (GRUM), Groupe de Recherche sur le Système Nerveux Central (GRSNC), Université de Montréal, Montréal, QC, Canada; ^8^Centre Interdisciplinaire de Recherche sur le Cerveau et l'Apprentissage (CIRCA), Université de Montréal, Montréal, QC, Canada; ^9^Centre de Recherche de l'Institut Universitaire de Gériatrie de Montréal (CRIUGM), Montréal, QC, Canada

**Keywords:** heart, brain, connection, dementia, Alzheimer's

## Introduction

The heart-brain axis to explain cognition, emotions, and the development of dementia remains largely unknown. Conversely, the influence of the brain on the heart, especially in old age also needs to be further explored.

Therefore, we initiated this Research Topic which includes six original and four review papers. They complemented each other, showing the influence the cardiovascular system has on the brain. Our goal was to provide insights into the possible psychological, social, and biological factors involved in the heart-brain interactions that can contribute to sickness and health. The manuscripts examined the pathophysiological impacts of high blood pressure, arterial stiffness, and the influence of heart function on brain and cognition. Two manuscripts also examined the involvement of the autonomic nervous system on the heart-brain connection. Studies presented in this Research Topic suggest that hypertension might not be directly responsible for Alzheimer's disease (AD), but that cerebrovascular damage reduces resistance to cognitive impairment and Alzheimer's disease.

## Blood and pulse pressures as risk factors

It is well-known that blood pressure changes over time are related to age, and that high blood pressure in people aged 50–70 is a risk factor for dementia later in life. Hypertension is also the most important risk factor for stroke. Selbaek et al. have evaluated blood pressure in older people over a period of 35 years with four measurements [1984–86 (HUNT1 study), 1995–97 (HUNT2), 2006–08 (HUNT3), and 2017–19 (HUNT4)]. The aim of this study was to compare trajectories of systolic blood pressure (SBP) in people with and without a diagnosis of dementia at the time of the fourth SBP measurement. At the 4th time point, 9,720 participants in a community survey were assessed. Compared to those without dementia, the participants with dementia had higher systolic blood pressure (SBP) at the first and second measurement, but lower SBP at the last measurement. The differences at first and second measurement compared with the last measurement were especially pronounced among women. Regarding dementia subtypes, those with vascular dementia had a higher SBP than those with the Alzheimer's type of dementia at the second and third measurement. Age trajectories in SBP showed that the dementia group experienced a steady increase in SBP until about 65 years of age and a decrease from 70 to 90 years. SBP in the group without dementia increased until 80 years before it leveled off from 80 to 90 years. Thus, it might be “normal” to have a steady raise in SBP until the age 80 before it levels off. However, those who had the highest blood pressure earlier were more likely to have a dementia diagnosis 10 years later. The results point to the importance of treating high blood pressure early in life. Earlier studies have indicated that the brain may adjust to the raise in pressure, but if a subsequent fall in SBP is too big, it may accelerate brain damage. A sudden and big SBP drop itself may be harmful to the brain. However, the cause-effect relationship is still uncertain. Does a significant drop in blood pressure create the brain damage, or does the brain damage create the blood pressure drop? With an increase in vascular pathology in the brain, manifestations of Alzheimer's disease can increase. The brain of a person with longstanding hypertension may therefore be more vulnerable to and have less reserves to compensate for development of Alzheimer's disease pathologies. Hypertension does not cause Alzheimer's disease, but may reduce the brain reserves the individual needs to function as a healthy person. Alzheimer's disease develops over years and does not manifest before the brain capacity is reduced to a point where it cannot compensate for the underlying pathology anymore.

Badji, Pereira et al. compared brain structure, cognitive performances, and cerebrospinal fluid (CSF) biomarkers of Alzheimer's disease and neurodegeneration, between normotensive and hypertensive (controlled, uncontrolled, and untreated) in a cohort of 70-year-old adults. The aim of this study was to examine whether controlling hypertension exerts beneficial effects on the brain. They found more white matter pathology (lower fractional anisotropy, more white matter hyperintensities, and enlarged perivascular space) in hypertensive individuals compared to their normotensive counterparts (highest in the uncontrolled participants). No significant difference was found in MRI or CSF markers of Alzheimer's disease pathology when normotensives were compared with hypertensive participants, nor among the hypertensive groups. This supports the fact that hypertension is not responsible for Alzheimer's disease, but, highly responsible for cerebrovascular damages leading to reduced brain reserves to withstand development of plaques and tangles that are responsible for dementia of the Alzheimer's type.

To understand the pathomechanisms by which vascular dysfunctions alter brain integrity, we need to know which parameters in the vascular system impact the brain's function. In a second contribution to this Research Topic, Badji, Cohen-Adad et al. studied the relationship between arterial stiffness index (ASI) measured with infrared light (photoplethysmography), pulse pressure (PP), and MRI markers of white matter integrity in 17,984 participants aged 63.09 ± 7.31 years from the UK Biobank. They concluded that brachial PP is a better predictor of white matter integrity than ASI. This was seen in those individuals younger than 75 years. No significant relationship was found between neither peripheral PP nor ASI and white matter integrity in the individuals older than 75 years of age. Comparing these findings with those reported by Selbaek et al., there seems to be an evidence for a shift at the age of about 75–80 regarding the relationship between vascular dysfunctions and cognition. One possible explanation is that a decline in blood pressure happens as a result of brain damage after the age of 75. This important decline in BP can further damage cerebrovascular integrity leading to dementia.

## Pathological mechanisms of the heart impacts the brain

It is known that areas around the hippocampus usually are the first ones to be involved in the neuropathological processes in Alzheimer's disease. It is also known that the hippocampus is very sensible to oxygen deficiency due to circulation failure, as often seen in overdoses of heroin. Niu et al. examined neural connectivity by analyzing MRI degree centrality in the hippocampus of patients with coronary artery disease (CAD). They found, in the CAD patients, a reduced connectivity in the right hippocampus, the right lingual gyrus and the left middle frontal gyrus. These observations correlated with the cognition scores. Niu et al. concluded that reduced cerebral neural connectivity may contribute to the cognitive impairment in CAD patients. The next manuscripts also examined the influence of heart pathology and cognitive deficits.

In a review, Myers et al. underline that atrial cardiopathy (a structural and functional disorder of the left atrium that may manifest as atrial fibrillation or heart failure) may contribute to cognitive impairment. This is an important Research Topic that has gained much interest in the literature and clinical practice especially because arterial cardiopathy can cause stroke.

Hagberg et al. summarized knowledge regarding consequences of out-of-hospital cardiac arrest (OHCA) on the brain. This is clearly in line with how oxygen deficiency to the brain may influence cognitive performance. After successful resuscitation, concerns regarding acquired brain injury and its sequels on cognitive functioning are relevant. Diffuse cortical and deep gray matter lesions are the most common findings with neuroimaging. Cognitive domains affected by OHCA are specifically executive functions, memory, and processing speed. Nevertheless, cognitive functions of OHCA survivors are not routinely assessed, and there is a lack of consensus that screening methods for cognitive changes should be applied. However, the authors emphasize that there is a need for an appropriate assessment as up to 50% of OHCA survivors develop cognitive decline.

## The autonomic nervous system: A cornerstone in the heart-brain connection

The autonomic nervous system is involved in the heart-brain connection. The two next studies looked at different aspects of the results of the autonomic nervous systems influence of the heart. Dolphin et al. reviewed the research to date investigating the cognitive effects of a non-invasive transcutaneous (t-VNS) device, and its impact on neuro-cardiovascular stability. How do t-VNS devices offer equivalent therapeutic potential as invasive devices without the surgical risks? They conclude that t-VNS shows promise as a neuro-modulatory technique in cognitive decline, and this may be *via* its ability to regulate both cardiac autonomic functions and to enhance cerebral perfusion.

Nicolini et al. studied heart rate variability as a predictor of cognitive decline in subjects with normal cognition or Mild Cognitive Impairment (MCI). This was a longitudinal study with ~3-year follow-up of a previous cross-sectional study. The participants were outpatients aged ≥ 65 when enrolled. They found that MCI patients had a greater response to a sympathetic challenge at baseline, related to steeper decline in episodic memory. A higher response to a parasympathetic challenge predicted a lesser decline in executive functioning. Thus, these intriguing results point to the impact of the autonomic nervous system on cognition.

## Depression: The cardiovascular link

Hakim addressed interconnections between depression and dementia. It is common knowledge that depression is related to shorter life expectancy, and that older people who suffer from depression have a higher risk than others to develop dementia in the future. People with depression usually die from the same diseases as others, mainly of cardiovascular diseases. It is also known that several people with psychiatric illnesses, including depression have an unhealthy lifestyle including sedentary lifestyle and smoking. The conclusion of the review is that depression may activate pro-inflammatory mediators, which may lead to cerebral small vessels impairment, with a consequent reduction in cerebral blood flow, causing cognitive deficits. Inflammation is a Research Topic where a lot has been done related to depression and heart disease. There are also data indicating a role for inflammation in dementia.

## Diabetes and sex-related brain vulnerability

Thomas et al. examined how diabetes is associated with brain structure and function in geriatric patients. They examined 893 patients (50% female) with MRI or CT scan and a thorough neuropsychological examination. They found that diabetes was associated with increased incidence of cerebral lacunes and brain atrophy in women compared to men. Thus, this study further supports differences in pathophysiological processes between men and women as seen in the study of Selbaek et al..

Taken together, the presented manuscripts bring further evidence for a close connection between the cardiovascular and the central nervous system and shed light on some of the potential mechanisms. Knowledge gained from these studies contributes to develop preventive approaches to protect two vital systems, including the reduction of their dysfunctional interactions.

## Author contributions

All authors listed have made a substantial, direct, and intellectual contribution to the work and approved it for publication.

## Conflict of interest

The authors declare that the research was conducted in the absence of any commercial or financial relationships that could be construed as a potential conflict of interest.

## Publisher's note

All claims expressed in this article are solely those of the authors and do not necessarily represent those of their affiliated organizations, or those of the publisher, the editors and the reviewers. Any product that may be evaluated in this article, or claim that may be made by its manufacturer, is not guaranteed or endorsed by the publisher.

